# Individual Differences in the Attribution of Incentive Salience to a Pavlovian Alcohol Cue

**DOI:** 10.3389/fnbeh.2016.00238

**Published:** 2016-12-26

**Authors:** Franz R. Villaruel, Nadia Chaudhri

**Affiliations:** Center for Studies in Behavioral Neurobiology/Groupe de Recherche en Neurobiologie Comportementale, Department of Psychology, Concordia UniversityMontreal, QC, Canada

**Keywords:** sign-tracking, goal-tracking, incentive salience, autoshaping, conditioned reinforcement, Pavlovian conditioning, alcohol, individual differences

## Abstract

Individual differences exist in the attribution of incentive salience to conditioned stimuli associated with food. Here, we investigated whether individual differences also manifested with a Pavlovian alcohol conditioned stimulus (CS). We compiled data from five experiments that used a Pavlovian autoshaping paradigm and tests of conditioned reinforcement. In all experiments, male, Long-Evans rats with unrestricted access to food and water were acclimated to 15% ethanol. Next, rats received Pavlovian autoshaping training, in which a 10 s presentation of a retractable lever served as the CS and 0.2 mL of 15% ethanol served as the unconditioned stimulus (US). Finally, rats underwent conditioned reinforcement tests in which nose-pokes to an active aperture led to brief presentations of the lever-CS, but nose-pokes to an inactive aperture had no consequence. Rats were categorized as sign-trackers, goal-trackers and intermediates based on a response bias score that reflected their tendencies to sign-track or goal-track at different times during training. We found that distinct groups of rats either consistently interacted with the lever-CS (“sign-trackers”) or routinely approached the port during the lever-CS (“goal-trackers”) across a majority of the training sessions. However, some individuals (“shifted sign-trackers”) with an early tendency to goal-track later shifted to comparable asymptotic levels of sign-tracking as the group identified as sign-trackers. The lever-CS functioned as a conditioned reinforcer for sign-trackers and shifted sign-trackers, but not for goal-trackers. These results provide evidence of robust individual differences in the extent to which a Pavlovian alcohol cue gains incentive salience and functions as a conditioned reinforcer.

## Introduction

Environmental stimuli that are associated with the reinforcing effects of drugs of abuse can acquire incentive motivational properties that drive drug-seeking and drug-taking behaviors (Stewart et al., 1984; Robinson and Berridge, 1993). In preclinical models, incentive stimuli are characterized by their ability to elicit approach, invigorate instrumental responding and function as conditioned reinforcers (Cardinal et al., 2002; Milton and Everitt, 2010). The ability of incentive stimuli to elicit approach is often indexed using a Pavlovian autoshaping paradigm (Brown and Jenkins, 1968; Hearst and Jenkins, 1974) in which a discrete conditioned stimulus (CS) is repeatedly paired with an appetitive unconditioned stimulus (US). Over repeated pairings, subjects can develop a “sign-tracking” conditioned response, which consists of approach and contact with the CS. These behaviors occur despite a lack of contingency between sign-tracking and US delivery (Hearst and Jenkins, 1974). The development of sign-tracking is indicative of the CS having gained incentive salience, a property that may enable the development and maintenance of addictive behaviors (Stewart et al., 1984; Robinson and Berridge, 1993).

Pavlovian cues that are associated with drugs of abuse elicit sign-tracking responses. For example, rats will sign-track to discrete cues that are paired with cocaine (Uslaner et al., 2006), opioids (Yager et al., 2015) and alcohol (Krank, 2003; Tomie et al., 2003; Krank et al., 2008; Srey et al., 2015). Studies showing sign-tracking with a CS that predicts alcohol (alcohol CS) sometimes employ food deprivation and/or sweetened ethanol solution (Krank, 2003; Tomie et al., 2003), both of which can artificially boost the attribution of incentive salience to the alcohol CS. Our laboratory recently showed that a CS paired with unsweetened ethanol solution supported sign-tracking in non-deprived rats and also functioned as a moderate conditioned reinforcer (a CS that is capable of facilitating the acquisition of a novel operant response; Srey et al., 2015). Thus, Pavlovian cues that predict alcohol can become imbued with incentive motivational properties.

However, there is also substantial evidence of individual differences in the attribution of incentive salience to appetitive Pavlovian cues (Robinson and Flagel, 2009; Meyer et al., 2012a). In contrast to sign-tracking, some animals develop a “goal-tracking” conditioned response, consisting of approach to the location where the US is delivered (goal-trackers; Boakes, 1977; Robinson and Flagel, 2009). Interestingly, it is only in individuals that develop a sign-tracking response (sign-trackers) that a food CS also subsequently functions as a conditioned reinforcer (Robinson and Flagel, 2009). The propensity to attribute incentive salience to a discrete food CS has been linked to vulnerability to the behavioral effects of discrete drug-associated cues (Flagel et al., 2009). For example, relative to goal-trackers, rats previously identified as sign-trackers with a food CS also subsequently attributed incentive salience to discrete conditioned cues associated with cocaine (Meyer et al., 2012b; Yager and Robinson, 2013), or an opiate (Yager et al., 2015), and were more likely to reinstate cocaine self-administration induced by discrete cues (Saunders and Robinson, 2010). In contrast, goal-trackers were more susceptible to the capacity of drug-associated contexts to trigger relapse (Saunders et al., 2014).

Drugs of abuse can influence and bias the development of conditioned approach responses. For example, pre-exposure to nicotine or amphetamine can bias responding towards sign-tracking, as well as invigorate the sign-tracking conditioned response (Doremus-Fitzwater and Spear, 2011; Palmatier et al., 2013). Recent work has also demonstrated that prolonged intermittent access to cocaine self-administration can increase motivation for cocaine to a similar extent in both sign-trackers and goal-trackers (Kawa et al., 2016). Similarly, we have reported that with extended training, an alcohol CS can eventually come to elicit sign-tracking, despite an early tendency for it to trigger goal-tracking (Srey et al., 2015). This shift in behavior from goal-tracking to sign-tracking may be a result of extended exposure to alcohol during Pavlovian training, which may facilitate the attribution of incentive salience to the alcohol CS. However, the extent to which individual subjects display this shift is not known.

The objective of the present study was to better characterize individual differences in the attribution of incentive salience to a Pavlovian alcohol CS. For this, we compiled data from five experiments that used a Pavlovian autoshaping paradigm in which non-deprived rats were trained to associate a discrete, localizable CS with unsweetened, 15% ethanol. A propensity to sign-track or goal-track was determined for individual rats using response bias scores in early, middle and late blocks of training. We report evidence for individual differences in the attribution of incentive salience to a Pavlovian alcohol CS, and show that in a subset of subjects the conditioned approach response shifts from goal-tracking to sign-tracking with extended training. Lastly, we show that a Pavlovian alcohol CS can function as a conditioned reinforcer in rats that either have a long history of sign-tracking or have shifted from goal-tracking to sign-tracking.

## Materials and Methods

### Subjects

Adult, male Long-Evans rats (Envigo, Indianapolis, IN, USA; 220–240 g on arrival; *N* = 107) from five different experiments were included in the present study. All rats were single housed in polycarbonate shoe-box cages (44.5 cm × 25.8 cm × 21.7 cm) containing beta chip bedding (Sani Chips, Envigo). Rats were acclimated to a controlled colony room for 1 week prior to experimental procedures (21°C; 44% humidity; 12 h light/dark cycle; lights on at 7:00 AM). All procedures were conducted during the light phase. Rats were provided with either a nylabone toy (Nylabones, Bio-Serv) alone or alongside a polycarbonate tunnel (Rat Retreats, Bio-Serv). Food (Agribands, Charles River) and water were unrestricted throughout the experiments. Experiments were conducted over a span of 4 years (2013–2016) by three different experimenters. Data from one the five experiments was previously published (Srey et al., 2015). All procedures are in accordance with the guidelines of the Canadian Council on Animal Care and approved by the Concordia University Animal Research Ethics Committee.

### Apparatus

Behavioral procedures were conducted in conditioning chambers (ENV-009A; Med Associates Inc., St-Albans, VT, USA) housed inside ventilated, sound-attenuating cubicles. Chambers were equipped with a house light (75W, 100 mA, ENV-215M) and consisted of a polycarbonate door, back wall and ceiling with stainless steel side walls, rod floors (ENV-009A-GF) and removable waste pan. For Pavlovian autoshaping training, a dual cup fluid port (ENV-200R3AM) was located centrally on the right wall. Ethanol was delivered into the port through a polyethylene tube connected to a 20 mL syringe that was mounted on a syringe pump (PHM-100, 3.33 rpm) outside the cubicle. Disruption of infrared beams across the port opening was used to measure port entries. Stainless steel retractable levers (4.8 cm × 1.9 cm; ENV-112M) elevated 6.9 cm above the rod floor flanked the left and right sides of the port. A weight of 25 g on the lever was required to produce a recordable lever activation.

During conditioned reinforcement, the retractable levers were replaced with nose poke apertures (ENV-114BM) elevated 2.8 cm above the rod floor. Disruption of infrared beams across the nose-poke openings was recorded as an entry. The fluid port was replaced with the left retractable lever used during Pavlovian autoshaping training. A computer and Med PC-IV software controlled all experimental events and recorded behavioral measures.

### Home-Cage Ethanol Exposure

A 15% ethanol (v/v) solution was prepared by diluting a 95% ethanol solution with tap water. Rats were acclimated to 15% ethanol using a 24 h intermittent access, two-bottle choice procedure that produces high levels of ethanol consumption in outbred rats (Wise, 1973; Simms et al., 2008; Sparks et al., 2013). Rats were weighed and given access to 15% ethanol and water every other day in their home cage. Ethanol was presented in a 100 mL pre-weighed graduated cylinder, and water was presented in a 400 mL pre-weighed plastic bottle. Both containers were sealed with metal sipper tubes containing metal ball bearings to minimize spillage. After 24 h of access to ethanol and water, both containers were removed and weighed. For the following 24 h, ethanol cylinders were replaced with a similar 100 mL pre-weighed graduated cylinder containing water, and the water bottles were placed back on the cage lids. Thus, rats had access to two sipper tubes at all times.

To mitigate side preference, the location of ethanol and water were randomly selected for each session. To account for spillage, ethanol cylinders and water bottles were placed onto an empty cage, and weighed at the same time as those in the experimental cages. Differences in weight between sessions in the empty cages could be attributable to spillage or evaporation, and average spillage across sessions was subtracted from each rat’s ethanol and water consumption.

The difference in ethanol cylinder weights in the 24 h period was used to calculate ethanol intake (grams of ethanol consumed per kilogram of body weight) and ethanol preference (grams of ethanol solution consumed divided by grams of total fluid consumption). In three of the five experiments, rats received 12 sessions of ethanol acclimation. One experiment consisted of 16 sessions and one experiment had 19 sessions. To facilitate analyses, only the first 12 sessions from each experiment are analyzed and reported.

Rats that failed to consume more than 1.0 g/kg/24 h of 15% ethanol in early sessions were either given a sweetened ethanol solution (15% ethanol and 2% sucrose) or a lower concentration of ethanol (5% ethanol) to encourage ethanol consumption for 2–5 sessions. Importantly, all rats had access to 15% ethanol in their final two acclimation sessions. Ten rats that failed to consume greater than 1.0 g/kg/24 h by the last two sessions were dropped from subsequent analyses (Carnicella et al., 2014). Remaining rats were counterbalanced based on ethanol intake, ethanol preference and body weight, and assigned to either paired or unpaired groups for Pavlovian autoshaping training.

### Behavioral Procedures

#### Habituation

Rats were habituated to the testing room and conditioning chambers over three consecutive days. On day 1, rats were habituated to the behavior room in their home cage for 20 min. On day 2, rats were handled and weighed in the behavior room and then returned to the colony room. On day 3, after being handled and weighed, rats were placed into their designated conditioning chambers for 20 min. The houselights turned on after a 1 min delay and fluid port entries were recorded, but had no programmed consequence.

#### Pavlovian Autoshaping Training

In each session, rats were weighed and placed in the conditioning chambers. The house light turned on after a 2 min delay to signal the start of the session. A retractable lever served as the CS (lever-CS) and was presented synchronously to all rats for 10 s. The location of the lever-CS (left or right of the fluid port) was counterbalanced across rats. For the paired group, the pump was activated for 6 s to deliver 0.2 mL of 15% ethanol US for oral consumption immediately after the lever-CS was retracted. For the unpaired group, ethanol was delivered midway through the interval between two consecutive lever-CS trials. Thus, both groups equally received 12 trials of the lever-CS and the ethanol US. Lever-CS trials occurred on a 260 s variable time interval (i.e., either after 140 s, 260 s, 380 s) excluding the 6 s period of pump activation (Srey et al., 2015). A total of 2.4 mL of 15% ethanol US was delivered per session for each rat. Each session lasted an average of 61.2 min, and ethanol consumption was verified at the end of each session by visual inspection. Four rats failed to consume the ethanol US and were therefore excluded from analyses.

Training consisted of 24 sessions for one experiment, 25 sessions for another experiment and 27 sessions for three experiments. In order to facilitate statistical comparisons, only the first 24 training sessions were analyzed and reported. Further, 12 rats were trained 5 days a week from Monday to Friday (*n* = 8, paired; *n* = 4, unpaired), 22 rats were trained on alternating days of the week including weekends (*n* = 14, paired; *n* = 8, unpaired), 11 rats were trained 7 days a week (*n* = 7, paired; *n* = 4, unpaired), and the remaining 62 rats were trained on Monday, Wednesday and Friday of each week (*n* = 47, paired; *n* = 15, unpaired).

#### Test for Conditioned Reinforcement

Conditioned reinforcement was tested in four of the five studies examined herein. Thus, for this analysis the paired group consisted of 52 rats and the unpaired group consisted of 31 rats. Approximately 48 h after the last Pavlovian autoshaping session, rats were habituated to the nose poke apertures used in the conditioned reinforcement paradigm. In this session, house lights were turned on prior to placing the rats in the chambers. The session began when a nose-poke occurred in one of the apertures and lasted 10 min thereafter. If no response was made, the habituation session ended after 10 min. Responses into the left and right nose poke apertures were recorded but had no programmed consequence. One of the four experiments did not include a habituation session to the nose pokes (Srey et al., 2015). Results from the habituation session, as well as lever-CS location, and the number of lever-CS activations in the last two Pavlovian autoshaping sessions were used to counterbalance the location (left or right relative to the lever-CS) of the “active” nose-poke aperture for the conditioned reinforcement test.

Conditioned reinforcement tests occurred approximately 24 h after the habituation session. Rats were weighed and placed in the conditioning chambers with the house lights turned on. The test began after a response in the “active” aperture and lasted 30 min thereafter. If a rat failed to make an active nose-poke then the test was terminated after 60 min. All rats made an active nose-poke before 60 min. During the test, “active” nose-pokes resulted in a 2.5 s presentation of the lever-CS. Responses in the “inactive” nose-poke had no programmed consequence. The first three earned presentations of the lever-CS occurred on a fixed-ratio one schedule. Subsequently, a variable ratio two schedule of reinforcement took effect in which 1, 2 or 3 active nose-pokes were reinforced with the lever-CS based on a Latin-square design (see Olausson et al., 2004; Chaudhri et al., 2006; Löf et al., 2010). The latter schedule was implemented in order to minimize within-subject extinction and allow for four conditioned reinforcement tests to be conducted, approximately 24 h apart from each another. The nose-poke designated as “active” remained consistent across all four tests. Repeated tests were conducted to replicate previous work (Srey et al., 2015) and to determine the time course of effects (Guy and Fletcher, 2013).

### Data Analysis

Body weight (g), ethanol intake (g/kg/24 h; grams of ethanol consumed per kilogram of body weight) and ethanol preference (%; grams of ethanol solution consumed divided by grams of total fluid consumed in the same session) were measured during home-cage ethanol exposure. During Pavlovian autoshaping training, lever-CS activations were used as a measure of sign-tracking, and normalized CS port entries, consisting of port entries made during the CS minus port entries made 10 s immediately before the CS, were used as a measure of goal-tracking. Latency and probability of conditioned responses were assessed as additional, supportive measures of sign- and goal-tracking. Latency was calculated as time in seconds to the first lever-CS activation or first port entry during a lever-CS trial. Probability to activate the lever-CS was calculated as the number of trials in which the lever-CS was activated divided by the total number of trials. Conversely, probability to make a port entry during the lever-CS trial was calculated as number of lever-CS trials during which a port entry was recorded divided by the total number of trials. Additionally, US port entries were measured as port entries during the 6 s interval immediately after lever-CS retraction (paired group) or in the middle of the inter-trial-interval period (unpaired group) during which the pump was activated and ethanol was delivered into the fluid port. In order to determine the behavioral phenotype of individual rats we calculated response bias scores according to the following formula: (lever-CS activations − CS port entries)/(lever-CS activations + CS port entries; Meyer et al., 2012a). Response bias was calculated for each training session and then averaged across eight sequential training blocks denoted as either early (session 1–8), middle (session 9–16) or late (session 17–24). Although published studies with a food pellet US have used the Pavlovian Conditioned Approach (PCA) index to characterize individual differences (Meyer et al., 2012a), we used a response bias score as a more general measure of conditioned response tendency to capture changes in response vigor over time. Further, our studies differ from studies in which the PCA index was developed in that we use a liquid US. The nature of the US can affect both the vigor and topography of conditioned responding (Davey and Cleland, 1982), which in turn can influence the PCA index. Dependent variables in conditioned reinforcement tests included entries into the active and inactive nose-poke apertures and frequency of lever-CS activations.

Graphs were created using Prism 6 and statistical analyses were conducted using SPSS Statistics Version 21. Data were analyzed using either repeated measures ANOVA or one-way ANOVA. Violations of sphericity were amended using the Huynh-Feldt correction. *Post hoc* analyses were done with Bonferroni’s correction. All analyses were evaluated with an alpha value of 0.05.

## Results

### Acquisition of Conditioned Responding in Paired and Unpaired Groups

**Figure 1:**
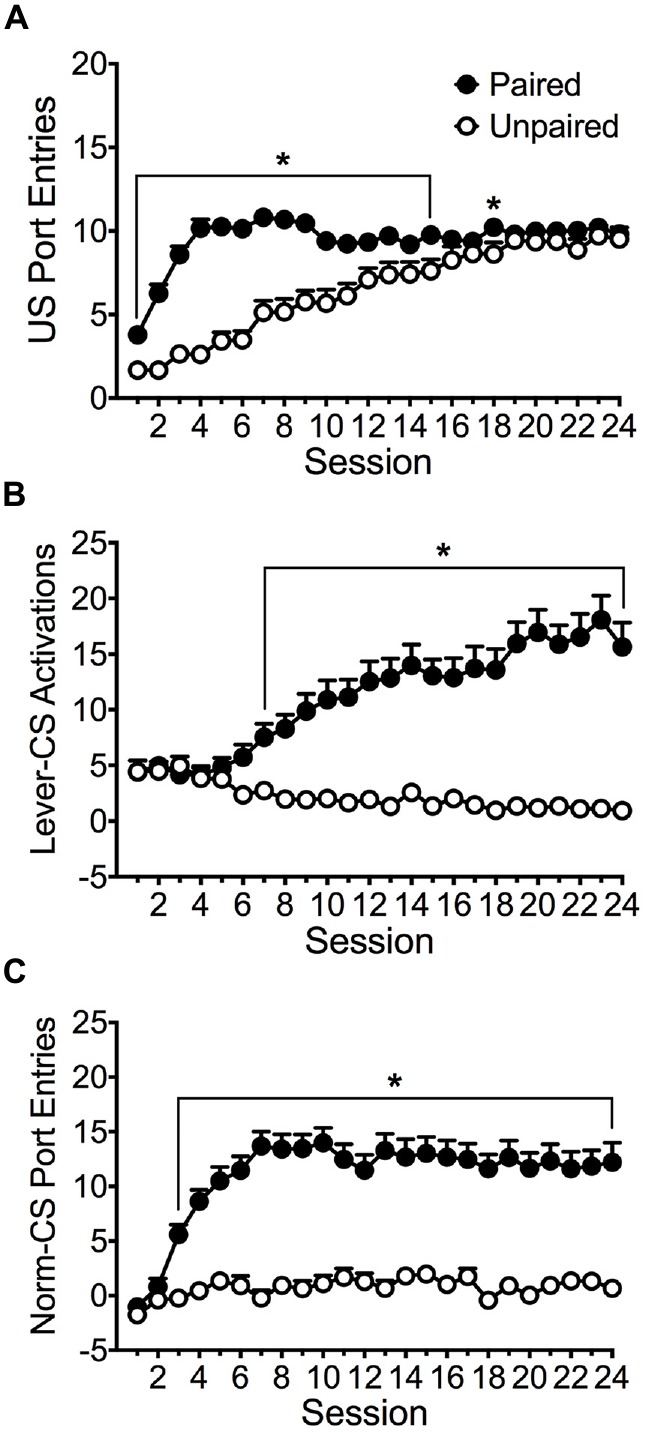
**Acquisition of responses across 24 Pavlovian autoshaping sessions.** Average (± SEM) **(A)** port entries made during the 6 s interval when the alcohol unconditioned stimulus (US) was delivered into the fluid port, **(B)** activation of the lever-CS during 10 s lever-CS trials, and **(C)** normalized entries into the fluid port during 10 s lever-CS trials. Data are normalized by subtracting port entries during 10 s intervals immediately before each CS from port entries during each CS. Black symbols represent the paired group (*n* = 76) and white symbols represent the unpaired group (*n* = 31). *p < 0.05, Bonferroni-corrected comparisons between paired and unpaired groups in each session.

Figure 1 depicts the acquisition of conditioned responding in paired (*n* = 76) and unpaired (*n* = 31) groups. The average port entries made during delivery of the ethanol US into the fluid port across 24 training sessions is shown in Figure 1A. Repeated measures ANOVA revealed significant main effects of Session (*F*_(23,2415)_ = 41.205, *p* < 0.001) and Group (*F*_(1,105)_ = 39.030, *p* < 0.001) and a significant Session × Group interaction (*F*_(23,2415)_ = 16.916, *p* < 0.001). Both groups learned to approach the port during ethanol delivery, but the paired group approached the port earlier in acquisition relative to the unpaired group. Bonferroni *post hoc* analysis revealed that the paired group made significantly more port entries than the unpaired group in sessions 1–15 (*p* < 0.05) and in session 18 (*p* = 0.023).

The paired group but not the unpaired group acquired sign-tracking, as measured using lever-CS activations (Figure 1B). Repeated measures ANOVA revealed significant main effects of Session (*F*_(23,2415)_ = 4.235, *p* = 0.001) and Group (*F*_(1,105)_ = 22.468, *p* < 0.001) and a significant Session × Group interaction (*F*_(23,2415)_ = 10.746, *p* < 0.001). Pairwise comparisons revealed that the paired group activated the lever-CS more than the unpaired group in sessions 7–24 (*p* < 0.05). A pairwise comparison of session 1 and 24 revealed that the paired (*p* < 0.001) but not the unpaired group (n.s.) acquired a sign-tracking conditioned response.

As depicted in Figure 1C, the paired group but not the unpaired group acquired goal-tracking, as measured using normalized CS port entries (CS port entries minus port entries during a 10 s pre-CS interval). Repeated measures ANOVA revealed significant main effects of Session (*F*_(23,2415)_ = 9.656, *p* < 0.001) and Group (*F*_(1,105)_ = 37.235, *p* < 0.001), and a significant Session × Group interaction (*F*_(23,2415)_ = 5.194, *p* < 0.001). Pairwise comparisons revealed that the paired group made more normalized port entries during lever-CS trials than the unpaired group in sessions 3–24 (*p* < 0.001). A pairwise comparison of session 1 and 24 confirmed that the paired group (*p* < 0.001) but not the unpaired group (n.s.) acquired a goal-tracking conditioned response.

### Characterization of Individual Differences in Conditioned Approach Responses

**Figure 2:**
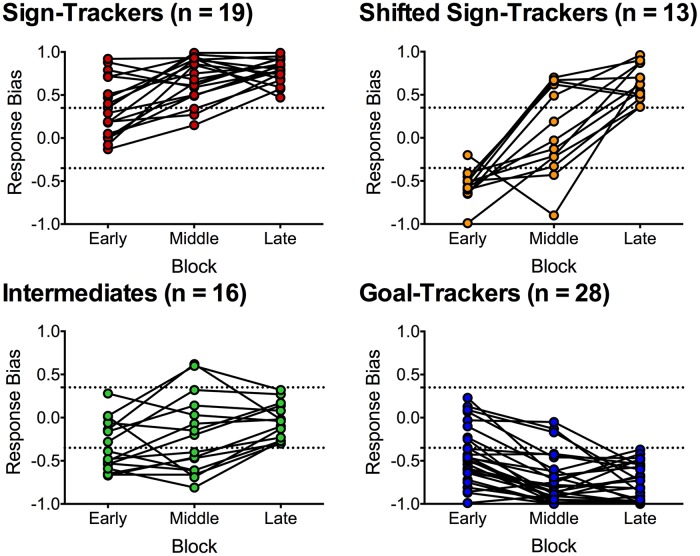
**Average response bias during an early (session 1–8), middle (session 9–16) and late block (session 17–24) of Pavlovian autoshaping training.** Response bias was calculated for each session by subtracting CS-elicited port entries from lever-CS activations and dividing by the sum of CS-elicited port entries and lever-CS activations, and then averaging across eight sessions. Individual rats were categorized based on the late block as either sign-trackers (*n* = 19), intermediates (*n* = 16) and goal-trackers (*n* = 28). Shifted sign-trackers (*n* = 13) were identified as having response bias scores comparable to sign-trackers in the late block and comparable to goal-trackers in the early or middle block. Dashed lines indicate response bias score cut-offs (0.35 and −0.35) for phenotype categorization.

Data from the 76 rats in the paired group were further analyzed using response bias scores to capture individual differences in the development of conditioned responses to an alcohol CS. Figure 2 depicts different phenotypes that emerged from categorizing individual rats in the paired group based on average response bias scores in early (session 1–8), middle (session 9–16) and late (session 17–24) blocks of training. Response bias was calculated as lever-CS activations minus CS port entries divided by lever-CS activations plus CS port entries. Thus, a positive value closer to 1 indicates a higher tendency to activate the lever-CS (sign-track), whereas a negative value closer to −1 indicates a higher tendency to make port entries during lever-CS trials (goal-track). Categorization into distinct phenotypes was based on behavior during the late training block. Rats with a response bias score between −1.00 to −0.36 were categorized as goal-trackers (*n* = 28), −0.35 to 0.35 were categorized as intermediates (*n* = 16), and 0.36 to 1.00 as sign-trackers (*n* = 19). A distinct population of sign-trackers was identified as shifted sign-trackers (*n* = 13) if they had response bias scores between 0.36 to 1.00 in the late block, but between −1.00 and −0.36 in either the early or middle training blocks.

### Potential Impact of Training Schedule on Phenotype

Intermittent exposure to drugs of abuse can induce incentive sensitization, which in turn might influence the attribution of incentive salience to appetitive Pavlovian cues and thereby impact the development of sign- and goal-tracking conditioned responses (Robinson and Berridge, 1993; Kawa et al., 2016). We therefore sought to determine if training schedule had a potential effect on phenotype distribution, bearing in mind the caveats that the numbers of rats within each training schedule were very different and that data for the present manuscript were obtained from experiments that had been conducted at different times and by different researchers.

Table 1 shows the distribution of rats within each phenotype in each of the four training schedules for the paired group. We conducted a chi-square test to determine if there was an association between training schedule and phenotype. The sample size assumption was violated (11 cells, 68.8%, had an expected count of less than 5), and therefore we report the likelihood ratio. This analysis revealed no significant association between phenotype and schedule (*X^2^_(9, N = 76)_* = 13.214, *p* = 0.153), suggesting that training schedule did not influence the phenotype that developed.

**Table 1:** **Distribution of phenotypes based on training schedule for rats in the paired group**.

	Mon-Wed-Fri	Alternating days	Mon-to-Fri	Everyday
Sign-tracker	9	4	2	4
Shifted sign-tracker	7	3	2	1
Intermediate	14	0	1	1
Goal-tracker	17	7	3	1
**Total (*n*)**	**47**	**14**	**8**	**7**

### Ethanol Intake and Preference of Different Phenotypes

**Figure 3:**
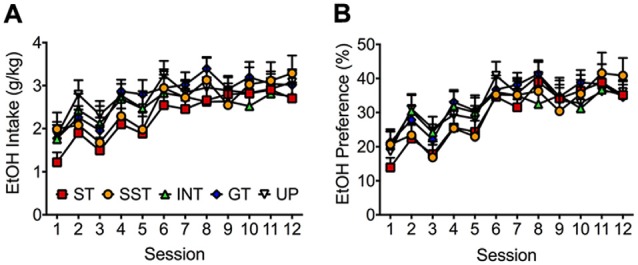
**Alcohol intake and preference increased for all phenotypes across 12 sessions of 15% alcohol exposure in the home-cage.** Average (± SEM) of **(A)** alcohol intake (g/kg) and** (B)** alcohol preference (%; grams of ethanol solution consumed divided by total fluid consumed) for paired rats categorized as sign-trackers (red; *n* = 19), shifted sign-trackers (yellow; *n* = 13), intermediates (green; *n* = 16) or goal-trackers (blue; *n* = 28), and unpaired rats (white; *n* = 31).

We evaluated if the different behavioral phenotypes were related to ethanol intake in the home cage prior to Pavlovian autoshaping training. Figure 3A depicts no significant difference in average 15% ethanol intake between the different phenotypes and the unpaired group across 12 sessions of home-cage ethanol exposure. Repeated measures ANOVA revealed a significant main effect of Session (*F*_(11,1122)_ = 17.805, *p* < 0.001), but no significant main effect of Group (*F*_(4,102)_ = 0.521, *p* = 0.720) or Session × Group interaction (*F*_(44,1122)_ = 0.895, *p* = 0.611). A paired samples *t*-test comparing session 1 and 12 of home-cage ethanol exposure revealed that ethanol intake increased across 12 session in all rats (*t*_(106)_ = 6.921, *p* < 0.001).

Ethanol preference was calculated as grams of ethanol solution consumed divided by total grams of fluid consumed in a single ethanol exposure session. As depicted in Figure 3B, there was no significant difference in preference for 15% ethanol between the different phenotypes and the unpaired group across 12 intermittent access sessions in the home-cage. Repeated measures ANOVA revealed a significant main effect of Session (*F*_(11,1122)_ = 23.077, *p* < 0.001), but no significant main effect of Group (*F*_(4,102)_ = 0.206, *p* = 0.935), or Session × Group interaction (*F*_(44,1122)_ = 0.814, *p* = 0.730). A paired samples *t*-test comparing session 1 and session 12 revealed an increase in ethanol preference across 12 sessions (*t*_(106)_ = 7.874, *p* < 0.001).

### US Port Entries of Different Phenotypes Across 24 Training Sessions

**Figure 4:**
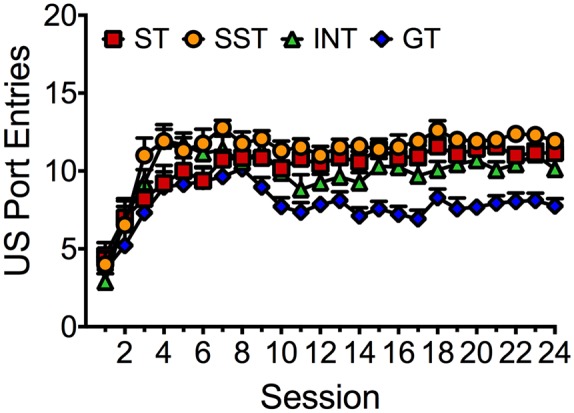
**Port entries made during ethanol delivery by each phenotype across 24 Pavlovian autoshaping sessions.** Data are expressed as average (± SEM) US port entries made by sign-trackers (red; *n* = 19), shifted sign-trackers (yellow; *n* = 13), intermediates (green; *n* = 16) or goal-trackers (blue; *n* = 28) in each session.

Figure 4 depicts average port entries made by the different phenotypes during the delivery of the ethanol US across 24 Pavlovian autoshaping sessions. Repeated measures ANOVA revealed significant main effects of Session (*F*_(23,1656)_ = 22.656, *p* < 0.001) and Phenotype (*F*_(3,72)_ = 19.051, *p* < 0.001), but no Session × Phenotype interaction (*F*_(69,1656)_ = 1.419, *p* = 0.087). Thus, all phenotypes learned to approach the port during delivery of the ethanol US. However, *post hoc* comparisons revealed that overall, goal-trackers made significantly fewer port entries than the intermediate phenotype (*p* < 0.001), sign-trackers (*p* < 0.001) and shifted sign-trackers (*p* < 0.001). A lower overall frequency of US port entries in goal-trackers would be observed if rats initiated a port entry during the CS and then remained in the port for the duration of US delivery. To verify that goal-trackers were not simply making fewer port entries *per se*, we analyzed port entries during the inter-trial interval (ITI), calculated as total port entries minus the sum of CS port entries and US port entries (data not shown). Repeated measures ANOVA revealed that ITI port entries decreased across Session (*F*_(23,1656)_ = 66.443, *p* < 0.001). However, there was no significant main effect of Phenotype (*F*_(3,72)_ = 1.951, *p* = 0.129) and no significant Session × Phenotype interaction (*F*_(69,1656)_ = 1.360, *p* = 0.084).

### Individual Variation in Conditioned Approach Responding Over 24 Training Sessions

**Figure 5:**
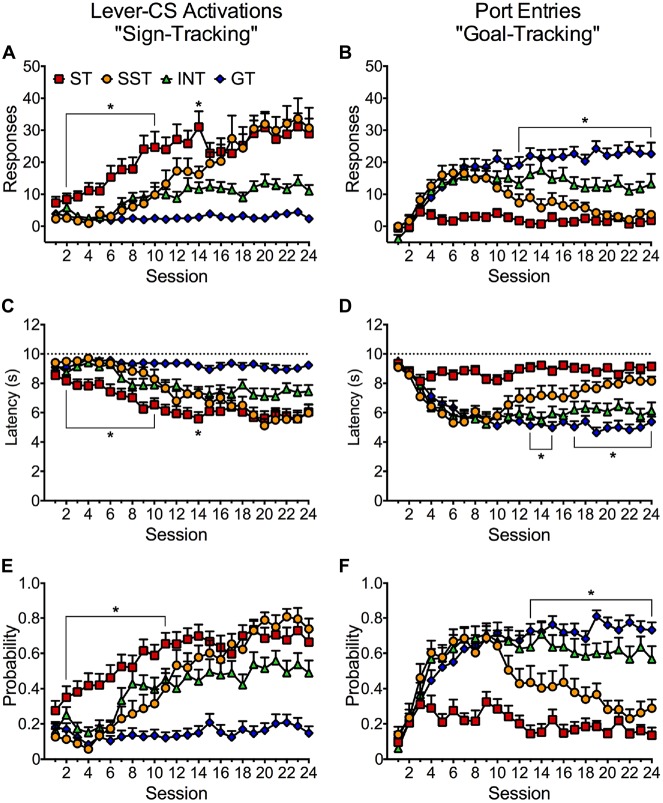
**Conditioned approach responses elicited by the lever-CS in four identified phenotypes across 24 sessions.** Data are expressed as average (± SEM) **(A)** lever-CS activations, **(B)** normalized port entries during lever-CS trials, **(C)** lever-CS activation latency, **(D)** CS elicited port entry latency, **(E)** probability to activate the lever-CS and **(F)** probability to make a port entry during a lever-CS trial. Dashed lines in **(C,D)** indicate the maximum latency to make a response or recorded latency if no response was made. *p < 0.05, Bonferroni-corrected comparisons of shifted sign-trackers relative to sign-trackers **(A,C,E)** and relative to goal-trackers **(B,D,F)** in each session.

Results from the phenotype analysis based on response bias scores were corroborated by an examination of the acquisition of lever-CS activations (sign-tracking) and normalized CS port entries (goal-tracking) across 24 Pavlovian autoshaping sessions (Figure 5). Figure 5A depicts distinct acquisition patterns for sign-tracking in each phenotype. A repeated measures ANOVA revealed significant main effects of Session (*F*_(23,1656)_ = 30.294, *p* < 0.001) and Phenotype (*F*_(3,72)_ = 29.832, *p* < 0.001), and a significant Session × Phenotype interaction (*F*_(69,1656)_ = 7.106, *p* < 0.001). Bonferroni corrected pairwise comparisons of session 1 and 24 revealed that rats classified as goal-trackers did not acquire a sign-tracking conditioned response (n.s.). The intermediate phenotype appears to have acquired sign-tracking but failed to reach statistical significance (n.s.). In contrast, sign-trackers (*p* < 0.001) and shifted sign-trackers (*p* < 0.001) both acquired a robust sign-tracking conditioned response. Pairwise comparisons revealed that shifted sign-trackers showed significantly higher lever-CS activations than the intermediate phenotype from session 17–24 (*p* < 0.05). Sign-trackers acquired the conditioned response much earlier in training than shifted sign-trackers. Further pairwise comparisons revealed that sign-trackers made significantly more lever-CS activations than shifted sign-trackers between sessions 1–10 (*p* < 0.05) and session 14 (*p* < 0.05). Shifted sign-trackers eventually reached the same level of lever-CS activations as sign-trackers in session 15 and onwards (n.s.).

Figure 5B depicts the acquisition patterns of normalized port entries (goal-tracking) for the four phenotypes across 24 Pavlovian autoshaping sessions. A repeated measures ANOVA revealed significant main effects of Session (*F*_(23,1656)_ = 18.379, *p* < 0.001) and Phenotype (*F*_(3,72)_ = 20.145, *p* < 0.001) and a significant Session × Phenotype interaction (*F*_(69,1656)_ = 7.272, *p* < 0.001). Bonferroni corrected pairwise comparisons of session 1 and 24 revealed that rats classified as sign-trackers did not acquire a goal-tracking conditioned response (n.s.). In contrast, both the goal-trackers (*p* < 0.001) and the intermediate phenotype (*p* = 0.001) acquired a goal-tracking response. A comparison of sessions 1 and 10 (*p* = 0.041) indicated that shifted sign-trackers acquired a goal-tracking response, but a comparison of sessions 1 and 24 (n.s.) indicated that this response later diminished. In later sessions, shifted sign-trackers reduced their goal-tracking response, whereas intermediates maintained moderate levels of goal-tracking. However, a comparison of the two phenotypes only revealed a significant difference at session 20 (*p* = 0.045). Further comparisons revealed that shifted sign-trackers were no different than goal-trackers in the frequency of normalized port entries in sessions 1–11 (n.s.). However, a significant difference emerged between goal-trackers and shifted sign-trackers in session 12 and onwards (*p* < 0.01), as the goal-tracking response diminished in shifted sign-trackers.

Figure 5C depicts the average latency to activate the lever-CS for the four phenotypes across 24 Pavlovian autoshaping sessions. Repeated measures ANOVA revealed significant main effects of Session (*F*_(23,1656)_ = 30.131, *p* < 0.001) and Phenotype (*F*_(3,72)_ = 27.812, *p* < 0.001), and a significant Session × Phenotype interaction (*F*_(69,1656)_ = 5.138, *p* < 0.001). In corroboration with the frequency of lever-CS activation (Figure 5A), Bonferroni corrected pairwise comparisons of sessions 1 and 24 revealed that the intermediate phenotype (n.s.) and goal-trackers (n.s.) did not become faster at activating the lever-CS, whereas sign-trackers (*p* = 0.001) and shifted sign-trackers (*p* < 0.001) became faster at activating the lever-CS after 24 sessions. Pairwise comparisons revealed that shifted sign-trackers were significantly faster at activating the lever CS than the intermediate phenotype in sessions 20, 22 and 23 (*p* < 0.05). Interestingly, lower latencies to activate the lever-CS were observed earlier in training in sign-trackers than in shifted sign-trackers. Pairwise comparisons revealed that sign-trackers activated the lever-CS faster than shifted sign-trackers in sessions 2–10 (*p* < 0.05) and session 14 (*p* = 0.03). Shifted sign-trackers activated the lever-CS at similar latencies to sign-trackers in session 15 and onwards (n.s.).

Figure 5D depicts the average latency to make a port entry during lever-CS trials for the four phenotypes across 24 Pavlovian autoshaping sessions. Repeated measures ANOVA revealed significant main effects of Session (*F*_(23,1656)_ = 18.189, *p* < 0.001) and Phenotype (*F*_(3,72)_ = 21.348, *p* < 0.001) and a significant Session × Phenotype interaction (*F*_(69,1656)_ = 6.031, *p* < 0.001). In support of normalized CS port entries (Figure 5B), Bonferroni corrected pairwise comparisons of session 1 and 24 revealed that sign-trackers (n.s.) did not become faster at entering the port during lever-CS trials. In contrast, both the intermediate phenotype (*p* < 0.001) and the goal-trackers (*p* < 0.001) had lower CS port entry latencies after 24 training sessions. Shifted sign-trackers also became faster at making CS port entries as seen by a pairwise comparison between sessions 1 and 11 (*p* = 0.012). However, a comparison of sessions 1 and 24 revealed that this reduced latency later diminished (n.s.). Pairwise comparisons revealed that the intermediate phenotype was significantly faster than shifted sign-trackers at making CS port entries in session 20, and in sessions 22–24 (*p* < 0.05), as goal-tracking diminished for shifted sign-trackers. Further analysis of the shifted sign-trackers revealed that they were no different than goal-trackers in latency to approach the port during lever-CS trials in session 1–12, and 16 (n.s.). However, a significant difference in latencies between shifted sign-trackers and goal-trackers emerged from session 13–15, and from session 17 and onwards (*p* < 0.05), as goal-tracking diminished in shifted sign-trackers but not in goal-trackers.

Figure 5E depicts the average probability to activate the lever-CS for the four phenotypes across 24 Pavlovian autoshaping sessions. Repeated measures ANOVA revealed significant main effects of Session (*F*_(23,1656)_ = 34.581, *p* < 0.001) and Phenotype (*F*_(3,72)_ = 36.586, *p* < 0.001), and a significant Session × Phenotype interaction (*F*_(69,1656)_ = 5.986, *p* < 0.001). In support of lever-CS activations (Figure 5A), Bonferroni corrected pairwise comparisons of session 1 and 24 revealed that goal-trackers (n.s.) did not increase in their probability to activate the lever-CS. In contrast, sign-trackers (*p* < 0.001), shifted sign-trackers (*p* < 0.001), and the intermediate phenotype (*p* = 0.024) all increased in their probability to activate the lever-CS after 24 sessions. Bonferroni corrected pairwise comparisons revealed that shifted sign-trackers were significantly more likely to make a lever-CS activation than the intermediate phenotype in sessions 20, 22 and 23 (*p* < 0.05). Sign-trackers were significantly more likely to make a lever-CS activation than shifted sign-trackers in sessions 2–11 (*p* < 0.05). However, probability to activate the lever-CS did not significantly differ between sign-trackers and shifted sign-trackers from session 12 and onwards (n.s.).

Figure 5F depicts the average probability to make a port entry during lever-CS trials for the four phenotypes across 24 Pavlovian autoshaping sessions. Repeated measures ANOVA revealed significant main effects of Session (*F*_(23,1656)_ = 22.851, *p* < 0.001) and Phenotype (*F*_(3,72)_ = 24.662, *p* < 0.001), and a significant Session × Phenotype interaction (*F*_(69,1656)_ = 7.454, *p* < 0.001). Pairwise comparisons of session 1 and 24 revealed that sign-trackers did not significantly increase in their probability to make a CS port entry. In contrast, both the intermediate phenotype (*p* < 0.001) and goal-trackers (*p* < 0.001) significantly increased in probability to make a CS port entry after 24 sessions. Shifted sign-trackers also increased in their probability to make a CS port entry as seen by a comparison between session 1 and 11 (*p* = 0.009). However, a pairwise comparison of session 1 and 24 revealed that probability to make a CS port entry later diminished (n.s.). Further analysis revealed that the intermediate phenotype was more likely than shifted sign-trackers to make a CS port entry on session 14, and from session 18 and onwards (*p* < 0.05), as goal-tracking diminished for shifted sign-trackers. Further analysis revealed that shifted sign-trackers did not significantly differ from goal-trackers in probability to make a CS port entry from session 1–12. However, as probability to make a CS port entry decreased for shifted sign-trackers a difference emerged relative to goal-trackers from session 13 and onwards (*p* < 0.05).

### Individual Variation in Conditioned Reinforcement

**Figure 6:**
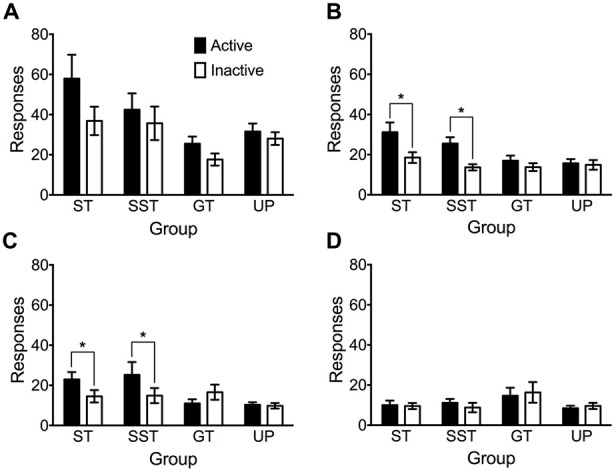
**Nose pokes made into the active and inactive apertures during tests of conditioned reinforcement.** Data are expressed as average (± SEM) responses into the active (black) and inactive (white) nose-poke apertures by each phenotype (sign-trackers, *n* = 13; shifted sign-trackers, *n* = 9; goal-trackers, *n* = 21) and the unpaired group (*n* = 31) during **(A)** test 1, **(B)** test 2, **(C)** test 3 and **(D)** test 4. *p < 0.05, Bonferroni-corrected comparisons.

Figure 6 depicts responding in active and inactive nose poke apertures in four conditioned reinforcement tests. The intermediate phenotype was removed from this analysis due to their vacillation between the sign- and goal-tracking conditioned responses in autoshaping sessions. An overall repeated measures ANOVA of the four tests revealed significant main effects of Test (*F*_(3,210)_ = 53.057, *p* < 0.001), nose poke Aperture (*F*_(1,70)_ = 13.966, *p* < 0.001), and Phenotype (*F*_(3,70)_ = 3.578, *p* = 0.018), but no significant Test × Aperture × Phenotype interaction (*F*_(9,210)_ = 1.553, *p* = 0.136). Due to the significant main effect of test, each conditioned reinforcement test was analyzed individually using repeated measures ANOVA.

There was no evidence of conditioned reinforcement in test 1 (Figure 6A). Analyses revealed significant main effects of Aperture (*F*_(1,70)_ = 13.173, *p* = 0.001), and Phenotype (*F*_(3,70)_ = 4.864, *p* = 0.004), but no significant Aperture × Phenotype interaction (*F*_(3,70)_ = 2.178, *p* = 0.098). *Post hoc* analysis revealed that overall, sign-trackers responded significantly more than goal-trackers (*p* = 0.003).

Conditioned reinforcement was observed in sign-trackers and shifted sign-trackers in test 2 (Figure 6B). ANOVA revealed significant main effects of Aperture (*F*_(1,70)_ = 16.330, *p* < 0.001) and Phenotype (*F*_(3,70)_ = 3.584, *p* = 0.018), and a significant Aperture × Phenotype interaction (*F*_(3,70)_ = 3.226, *p* = 0.028). Pairwise comparisons revealed that sign-trackers (*p* = 0.001) and shifted sign-trackers (*p* = 0.011) made significantly more nose pokes in the active aperture than the inactive aperture. In contrast, goal-trackers (*p* = 0.280) and the unpaired group (*p* = 0.753) failed to discriminate between active and inactive apertures.

Analysis of Test 3 revealed that the conditioned reinforcement effect persisted in both sign-trackers and shifted sign-trackers (Figure 6C). There was no significant main effect of nose poke Aperture (*F*_(1,70)_ = 3.224, *p* = 0.077), but a significant main effect of Phenotype (*F*_(3,70)_ = 4.662, *p* = 0.005) and Aperture × Phenotype interaction (*F*_(3,70)_ = 3.766, *p* = 0.014). Pairwise comparisons revealed again that sign-trackers (*p* = 0.042) and shifted sign-trackers (*p* = 0.037) made significantly more nose pokes in the active than the inactive aperture. Goal-trackers (*p* = 0.082) and the unpaired group (*p* = 0.854) did not discriminate between the two apertures.

By test 4 the conditioned reinforcement effect had diminished in both sign-trackers and shifted sign-trackers (Figure 6D). There were no main effects of Aperture (*F*_(1,70)_ = 0, *p* = 1.00) or Phenotype (*F*_(3,70)_ = 1.472, *p* = 0.230) and no Aperture × Phenotype interaction (*F*_(3,70)_ = 0.292, *p* = 0.831).

**Figure 7:**
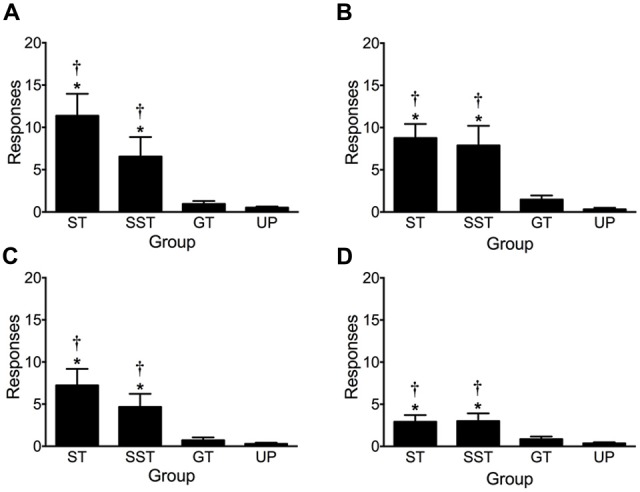
**Activation of earned presentations of the lever-CS during conditioned reinforcement tests.** Data are expressed as average (± SEM) lever-CS activations by each phenotype (sign-trackers, *n* = 13; shifted sign-trackers, *n* = 9; goal-trackers, *n* = 21) and the unpaired group (*n* = 31) during **(A)** test 1, **(B)** test 2, **(C)** test 3 and **(D)** test 4. *p < 0.05 Bonferroni corrected comparisons to the unpaired group and ^†^*p* < 0.05 to goal-trackers.

Figure 7 depicts lever-CS activations made during earned presentations of the lever-CS in the four tests of conditioned reinforcement. A repeated measures ANOVA revealed significant main effects of Test (*F*_(3,210)_ = 15.620, *p* < 0.001) and Phenotype, (*F*_(3,70)_ = 30.334, *p* < 0.001) and a significant Test × Phenotype interaction (*F*_(9,210)_ = 6.275, *p* < 0.001). Due to the significant effect of Test and Test × Phenotype interaction, each test was subsequently analyzed by a separate one-way ANOVA.

Across four tests of conditioned reinforcement, sign-trackers and shifted sign-trackers consistently activated the lever-CS more than goal-trackers and the unpaired group. In test 1, one-way ANOVA revealed a significant main effect of Group (*F*_(3,70)_ = 20.402, *p* < 0.001). This effect persisted across test 2 (Group, *F*_(3,70)_ = 22.676, *p* < 0.001), test 3 (Group, *F*_(3,70)_ = 15.286, *p* < 0.001) and test 4 (Group, *F*_(3,70)_ = 10.004, *p* < 0.001). *Post hoc* comparisons revealed that during test 1 sign-trackers (*p* < 0.001) and shifted sign-trackers (*p* = 0.006) activated the lever-CS significantly more than the unpaired group. Additionally, sign-trackers (*p* < 0.001) and shifted sign-trackers (*p* = 0.019) activated the lever-CS significantly more than goal-trackers. There was no difference in lever-CS activations between goal-trackers (n.s.) and the unpaired group. This pattern of results was also obtained in test 2 (ST vs. UP, *p* < 0.001; ST vs. GT, *p* < 0.001; SST vs. UP, *p* < 0.001; SST vs. GT, *p* < 0.001; GT vs. UP, n.s.), test 3 (ST vs. UP, *p* < 0.001; ST vs. GT, *p* < 0.001; SST vs. UP, *p* = 0.008; SST vs. GT, *p* = 0.031; GT vs. UP, n.s.), and test 4 (ST vs. UP, *p* < 0.001; ST vs. GT, *p* = 0.008; SST vs. UP, *p* = 0.001; SST vs. GT, *p* = 0.018; GT vs. UP, n.s).

## Discussion

The present study combined data from multiple experiments from our laboratory to investigate individual differences in the attribution of incentive salience to a Pavlovian alcohol CS. We found that a discrete Pavlovian CS that predicted alcohol acquired incentive motivational properties in some individuals but not others. Paired trials of a lever-CS with alcohol led to the acquisition of sign-tracking in some rats (sign-trackers) and goal-tracking in others (goal-trackers). These groups diverged early in autoshaping training and remained distinct across 24 sessions. Moreover, the lever-CS only functioned as a conditioned reinforcer in sign-trackers but not goal-trackers. We also found that a population of rats that could be characterized as sign-trackers at the end of training had an initially high tendency to goal-track. Had the experiments been concluded after 10 autoshaping sessions these rats would have been identified as goal-trackers. Interestingly, the lever-CS also functioned as a conditioned reinforcer in shifted sign-trackers, at comparable levels to that of sign-trackers. These behavioral phenotypes were not the result of different levels of alcohol intake in the home cage. The present findings show that individual differences exist in the attribution of incentive salience to a Pavlovian CS that predicts alcohol. The findings also suggest that with extended training, cues that predict alcohol may eventually acquire incentive motivational properties even in rats that do not initially have a propensity to attribute incentive salience to such cues.

The present findings on individual differences in the attribution of incentive salience to a Pavlovian alcohol CS are consistent with studies that use food as the US (Robinson and Flagel, 2009; Meyer et al., 2012a). Here, paired trials of a lever-CS with alcohol produced two distinct conditioned approach responses, one towards the lever-CS and another towards the site of alcohol delivery. Similar to a food CS, rats showed a bias towards one conditioned approach response over the other with an alcohol CS. Specifically, sign-trackers readily approached and activated the lever-CS, but goal-trackers approached the site of alcohol delivery during lever-CS trials. These two responses were acquired early in training and remained stable across 24 sessions for each phenotype. The frequency of responding was corroborated by lower latencies to activate the lever-CS in sign-trackers and lower latencies to enter the port in goal-trackers. As with a food CS (Boakes, 1977; Flagel et al., 2008), we also observed an intermediate phenotype that vacillated between both conditioned approach responses and did not reach the same asymptotic levels of responding as either sign- or goal-trackers.

Individual differences were also evident during tests of conditioned reinforcement for an alcohol CS. Specifically, sign-trackers but not goal-trackers discriminated between active and inactive nose pokes to earn presentations of the lever-CS that was previously paired with alcohol. However, this effect was only observed in the second and third conditioned reinforcement tests. Sign-trackers may not have expressed conditioned reinforcement in the first test because it was their first time experiencing the nose poke apertures and the operant contingencies. This claim is supported by evidence of higher overall responding in sign-trackers than goal-trackers in the first conditioned reinforcement test, suggesting that the lever-CS may have indeed been reinforcing operant responses but that the novelty of the test *per se* masked this effect. Failure to see conditioned reinforcement in the fourth test could be attributed to extinction effects, as alcohol was not present during these tests. Interestingly, sign-trackers but not goal-trackers activated the lever-CS when it was earned in all four conditioned reinforcement tests, despite failing to discriminate between active and inactive nose pokes apertures in tests 1 and 4. Thus, the lever-CS likely retained its incentive value even without proper expression of operant contingencies in tests 1 and 4. This dissociation in nose poke responding and lever-CS activation is consistent with the suggestion that conditioned approach and conditioned reinforcement may be distinct psychological constructs mediated by different neurobiological substrates (Cardinal et al., 2002).

Interestingly, the present study found that a group of rats that initially acquired a goal-tracking conditioned response could be identified as sign-trackers later in training. We have previously demonstrated a shift from goal-tracking to sign-tracking using the same procedure (Srey et al., 2015). By examining individual differences in a large sample of subjects consolidated across several studies, the present data replicate and extend our prior findings, showing that a shift from goal-tracking to sign-tracking occurs in a subset of individuals. Shifted sign-trackers were observed in all five experiments. This phenotype was behaviorally no different from rats identified as goal-trackers in sessions 1–11. However, with extended training goal-tracking responses diminished and were superseded by sign-tracking responses in these individuals. In fact, from session 15–24, rats that shifted to sign-tracking did so at comparable asymptotic levels as rats that acquired sign-tracking early in training (sign-trackers). Therefore, shifted sign-trackers are distinct from sign-trackers and goal-trackers in that they display both conditioned responses. Shifted sign-trackers are also distinct from the intermediate phenotype. Whereas the intermediate phenotype maintained sub-asymptotic levels of both sign- and goal-tracking responses, shifted sign-trackers displayed comparable levels of sign-tracking to sign-trackers and comparable levels of goal-tracking to goal-trackers at different times during training.

The lever-CS also functioned as a conditioned reinforcer in shifted sign-trackers. As in sign-trackers, shifted sign-trackers also learned to discriminate between active and inactive apertures to earn presentations of the lever-CS. Furthermore, despite failing to discriminate between the apertures in tests 1 and 4, shifted sign-trackers activated the lever-CS upon its presentation in all four tests. Thus, as was the case for sign-trackers, the lever-CS likely functioned as an incentive stimulus in all four conditioned reinforcement tests for shifted sign-trackers. Given that shifted sign-trackers also exhibited goal-tracking responses early in training, it would be of interest to see if these individuals would have shown conditioned reinforcement if they had been tested before acquiring the sign-tracking response. Overall, the observation of this shifted sign-tracking phenotype suggests that with extended exposure, cues that predict alcohol may ultimately acquire incentive motivational properties for some individuals.

A shift from goal-tracking to sign-tracking is in accordance with the incentive sensitization framework, which highlights that repeated exposure to addictive substances and their associated cues may lead to maladaptive attribution of incentive salience (Robinson and Berridge, 1993). In support of this hypothesis, we found no cases in which rats with an early tendency to sign-track later developed a goal-tracking response. Further, barring very few cases (*n* = 2) in which rats developed a sign-tracking response in the middle block and were later categorized as intermediates, the general trend with extended Pavlovian autoshaping sessions was to either maintain a specific conditioned response or shift from a goal-tracking to a sign-tracking response. However, others have found that conditioned responses elicited by a food CS are stable across 22 sessions of Pavlovian training (Robinson and Flagel, 2009). This suggests that neuroadaptations induced by pharmacologically active substances like alcohol might facilitate a switch in conditioned approach responses. For example, others have found that rats previously characterized as goal-trackers with a food CS can increase in their probability to sign-track when introduced to a new CS that predicts cocaine (Yager and Robinson, 2013). Additionally, after intermittent self-administration of cocaine, both goal- and sign-trackers display comparable increases in levels of motivation for the drug (Kawa et al., 2016). Further, an opiate (remifentanil) CS can also function as a conditioned reinforcer, albeit at a low dose in rats previously identified as goal-trackers with a food CS (Yager et al., 2015). However, characterizing a switch from goal-tracking to sign-tracking was not feasible in these studies because cocaine and remifentanil were delivered through an intravenous catheter: thus, goal-tracking could not be measured (Yager and Robinson, 2013; Yager et al., 2015). The use of alcohol as a reinforcer that is orally consumed presents the opportunity to study the trajectory of goal-tracking responses with a drug-associated CS, which has not been demonstrated with intravenous drug delivery.

A shift from goal-tracking to sign-tracking is also consistent with reports of procedures that sensitize the mesolimbic system to increase the tendency and augment the sign-tracking response. For example, stress in the form of early social deprivation (Lomanowska et al., 2011), reducing the certainty with which a CS predicts a reward (Anselme et al., 2013), and amphetamine sensitization (Doremus-Fitzwater and Spear, 2011; Robinson et al., 2015) have all been found to promote sign-tracking. We posit that another factor that could bias responding towards sign-tracking is extended autoshaping training with a CS that predicts a drug, as we demonstrate here with alcohol.

The shift from goal-tracking to a sign-tracking response is also supported by the neurobiological changes that occur with extended Pavlovian autoshaping training. For example, Tomie et al. (2000) showed higher expression levels of DOPAC (a dopamine metabolite) in the nucleus accumbens (NAc) during asymptotic performance of sign-tracking, compared to during acquisition. Further, the mRNA of D1 receptors in the NAc, which are implicated in the acquisition of sign-tracking (Dalley et al., 2005), have been found to increase in goal-trackers following Pavlovian training (Flagel et al., 2007). These neurobiological changes parallel findings that chronic exposure to alcohol can sensitize the mesolimbic dopamine system and that sensitized locomotor activation is modulated by D1 receptors in the NAc (Brodie, 2002; Abrahao et al., 2011). Thus, a sensitization of the dopamine system brought about by repeated, intermittent exposure to alcohol during Pavlovian autoshaping training in the present study may mediate the shift from goal-tracking to sign-tracking with extended training.

In conclusion, individual differences exist in the attribution of incentive salience to a Pavlovian CS that predicts alcohol. For sign-trackers, but not goal-trackers the CS promotes approach and interaction and can also serve as a conditioned reinforcer. We also find that with extended training some goal-trackers ultimately attribute incentive salience to the CS and become sign-trackers. For these shifted sign-trackers the CS promotes approach and interaction later in training, but still serves as a conditioned reinforcer. More research is required to delineate the underlying neural mechanisms that are involved in the attribution of incentive salience to Pavlovian alcohol cues. Understanding these processes may lead to the prevention or management of maladaptive attribution of incentive salience to drug predictive cues, which could mitigate the propensity for relapse in drug addiction.

## Author Contributions

FRV collected the majority of the data, analyzed and interpreted the findings, and wrote the manuscript. NC helped in analyzing and interpreting findings and provided critical revisions to the manuscript. Both FRV and NC gave final approval of the version to be published and agree to be accountable for all aspects of the work.

## Conflict of Interest Statement

The authors declare that the research was conducted in the absence of any commercial or financial relationships that could be construed as a potential conflict of interest.
